# An example of governance for AI in health services from Aotearoa New Zealand

**DOI:** 10.1038/s41746-023-00882-z

**Published:** 2023-09-01

**Authors:** R. Whittaker, R. Dobson, C. K. Jin, R. Style, P. Jayathissa, K. Hiini, K. Ross, K. Kawamura, P. Muir, the Waitematā AI Governance Group, A. Mark, D. Armstrong, E. Frost, J. Buxton, J. Lunny, P. Andrew, S. Bloomfield, S. Puddle, W. Miles

**Affiliations:** 1 Te Whatu Ora Waitematā, Auckland, New Zealand; 2 National Institute for Health Innovation, University of Auckland, Auckland, New Zealand; 3 Precision Driven Health, Auckland, New Zealand

**Keywords:** Health services, Translational research

## Abstract

Artificial Intelligence (AI) is undergoing rapid development, meaning that potential risks in application are not able to be fully understood. Multiple international principles and guidance documents have been published to guide the implementation of AI tools in various industries, including healthcare practice. In Aotearoa New Zealand (NZ) we recognised that the challenge went beyond simply adapting existing risk frameworks and governance guidance to our specific health service context and population. We also deemed prioritising the voice of Māori (the indigenous people of Aotearoa NZ) a necessary aspect of honouring Te Tiriti (the Treaty of Waitangi), as well as prioritising the needs of healthcare service users and their families. Here we report on the development and establishment of comprehensive and effective governance over the development and implementation of AI tools within a health service in Aotearoa NZ. The implementation of the framework in practice includes testing with real-world proposals and ongoing iteration and refinement of our processes.

## Introduction

Artificial Intelligence (AI) in healthcare is undergoing a rapid development phase, with advances in research across many different medical fields^1^. The potential for improvements in diagnostic accuracy, efficiency, treatments, and patient outcomes are widely discussed^2–5^, but for many, that potential is yet to be realised. This is partly due to the risks and issues surrounding the implementation of AI tools in clinical practice^6–9^.

Whilst several governance frameworks and supporting guidance materials have been published^8,10–14^ minimal information has been published around the actual implementation of an AI governance body within a health service organisation. Further, there is little published that is specific to the Aotearoa New Zealand (NZ) context, beyond Te Pokapū Hātepe o Aotearoa, the New Zealand Algorithm Hub^15^.

We feel that the importance of ensuring a voice for healthcare patients and their families, and for Māori (the indigenous people of Aotearoa NZ) to address health inequities that are exacerbated and perpetuated through breaches of Te Tiriti (the Treaty of Waitangi), is not sufficiently recognised in international guidance or previous reports. We recognise that the challenge goes beyond simply modifying existing risk frameworks or governance guidance. It must encompass the country’s progress on addressing longstanding disparities and health inequities for Māori based on the principles of Te Tiriti and the findings of the Waitangi Tribunal Inquiry into Health Services and Outcomes (that is, the principles of tino rangatiratanga (self-determination and autonomy), equity, active protection, options and partnership)^16^. This is detailed in Whakamaua: Māori Health Action Plan 2020–2025^17^ with outcomes such as addressing racism in the health system, and in Pae Ora (Healthy Futures) Act 2021^18^ which lays the foundation for the transformation of the NZ health system. We can also learn from work by Hudson et al. in ‘Te Ara Tika’ which provides a framework for ensuring research is undertaken based on tikanga and matauranga Māori (Māori ethical principles and philosophies)^19^.

In this paper we describe the establishment of comprehensive governance over the development and implementation of AI tools within our health service (Te Whatu Ora Waitematā, a public health service responsible for the health of approximately 650,000 people in a geographical district of Auckland, Aotearoa NZ). Through sharing our experiences, highlighting that it is not as simple as adapting international guidance to a specific context and population, we hope to help ensure that health organisations planning to implement AI and those in the technology sector, develop AI tools in line with good public health governance processes.

## Results

### The framework

**Fig. 1:**
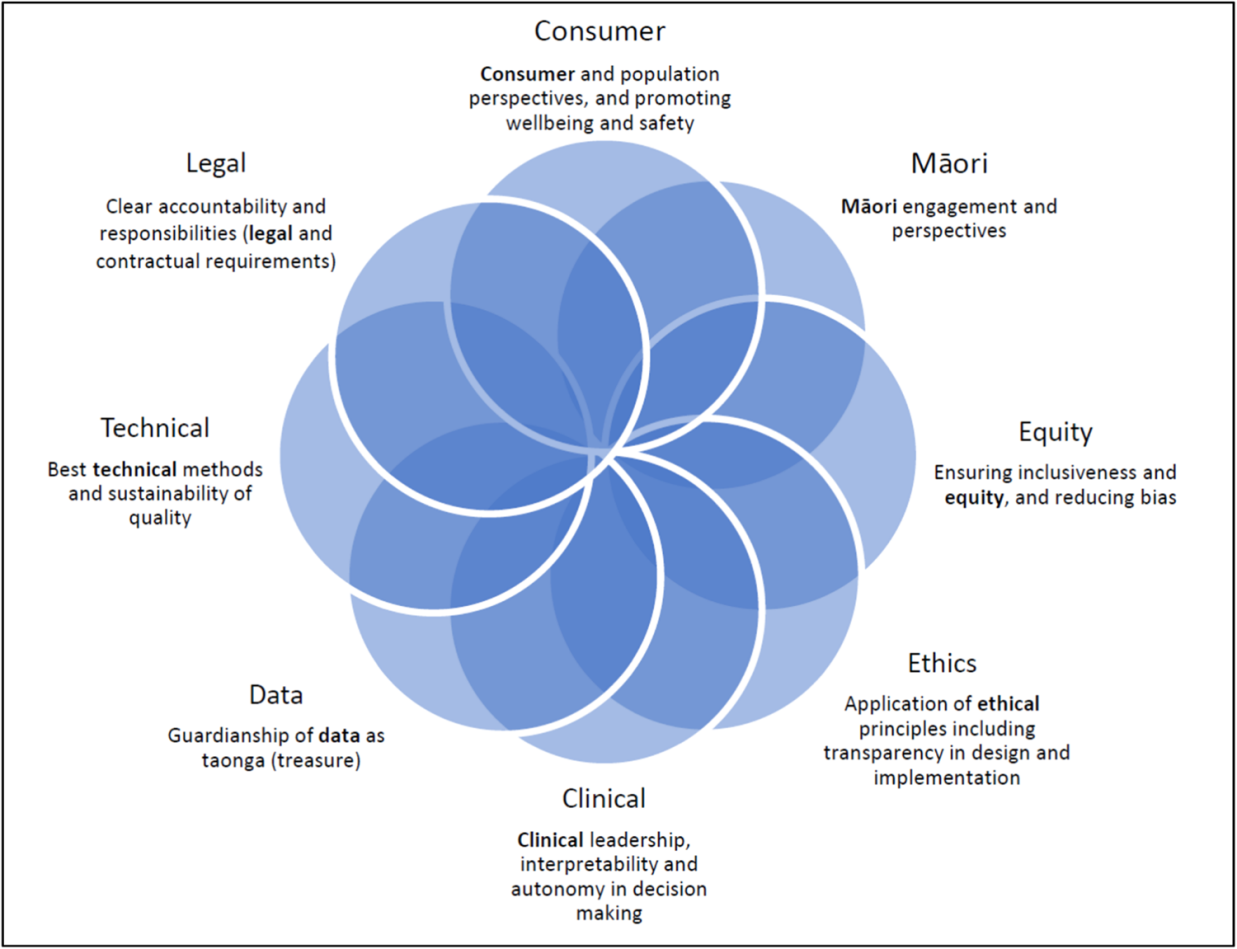
Domains for consideration. AI governance checklist: eight domains for consideration.

The full governance framework is shown in the Supplementary Information. The newly established Artificial Intelligence Governance Group (AIGG) agreed on one initial over-arching question with further considerations in eight domains (Fig. 1). The first question is whether it is appropriate to use AI in the proposed context. This is to ensure that the problem at hand is clearly defined and well understood. In addition, the data required for AI development or implementation needs to be explored to ensure that it is of sufficient scale and quality. If the data needed to fuel an AI is of poor quality or not easily accessible for any reason, then an AI based solution is unlikely to be worthwhile^13^.

Under each of the eight domains there are a range of questions adapted from international frameworks to fit both the Aotearoa NZ’s context and its health system’s context (Supplementary Information). These questions are framed according to the stage in the lifecycle that the AI project is proposed, classified into the following stages:

1. A new concept to be explored
2. At the point of requesting access to data for pre-processing or model development
3. At the point of clinical validation and/or implementation into the health service

### The domains

*Consumer perspectives* are prioritised throughout the process to ensure our population would be comfortable with the use of AI in the context proposed. Proposals need to demonstrate that they understand the potential benefits, and perhaps more importantly, identify any potential risks for consumers in order to satisfy the consumer representation on the AIGG. Findings from the patient survey^20^, showed that there needed to be transparency and communication around what projects are using aggregated data. Therefore, ensuring that there is clear public communication will, in part, be the responsibility of the AIGG as well as the service intending to develop or implement the models.

*Māori perspectives* reflect the importance of Māori data as a taonga (a treasure) requiring Māori governance and partnership as outlined in the Te Mana Raraunga Principles of Māori Data Sovereignty^21^. This includes questions around the involvement of Māori world perspectives, as well as Māori developers/clinicians/patients/communities in the development of AI, and the ability of the team to design and develop the AI in a culturally appropriate manner. The principles for Māori Data Sovereignty that need to be embedded in governance of AI include consideration of individual and collective benefits, and respect for Māori knowledge and protocols. The inclusion of Māori perspectives in the AIGG also encourages Te Tiriti based arrangements, ensuring acceptability and accountability to Māori with a commitment to equitable benefits through a sustained focus on mana (mutual respect).

*Equity and fairness* particularly focus on the likelihood of any bias or discrimination issues that may exist in the data collected or are unintentionally introduced by the model. In order to address these risks, good model developers should employ strategies which mitigate bias during model development as well as testing for bias once developed. This can only be done with local understanding of the intent and processes for data collection, changes in clinical practice over time in our health services, and recognition of the existing inequities in access and health outcomes for Māori and other groups that may be reflected in the data.

*Ethical principles* embedded in the design and development of the AI should be clear and include consideration of the New Zealand National Ethical Standards^22^. In particular, the AIGG checklist includes transparency as a key principle, and this is reflected throughout the various stages of the AI lifecycle. Transparency for this purpose includes sharing of information about data used, methodology for model development, processes around model validation and outcome testing, model features and potentially the algorithm itself. A high level of transparency is required to build trust amongst clinicians ensuring any decisions based upon model outputs are well understood, safe and accurate. We also consider that clinical practice in Aotearoa NZ is not ready for fully ‘black box’ algorithms but rather clinicians, in general, require an understanding of the process and the important factors in any algorithm. Furthermore, whilst AI may influence the decision-making process, key clinical decisions will continue to be made by clinicians for the foreseeable future in our health services in order to ensure that human oversight, judgement and evaluation are involved. This is in line with the principles outlined by Office of the Privacy Commissioner and StatsNZ^23^.

*Clinical perspectives* reflect the involvement of clinicians in the concept and development phases, their comfort with the evidence of accuracy, and how easily the AI can fit within existing clinical workflows. Without an understanding of how local health services currently work, it is entirely possible for new developments to completely miss the mark in assisting them, even as newer ways of working emerge. The AIGG checklist includes the necessary involvement of clinicians or clinical services from the beginning.

*Data issues* are wide ranging but include whether there is sufficient data that is both complete and representative of the full population group affected. If data from any groups are missing for any reason, then bias will be introduced. Further conscious or unconscious biases may arise through the processes of collecting the data. Those involved must understand how the data is collected and recorded to ensure it is used in an appropriate way^24^. Developers must be prepared to be transparent about the data that was used for training (including how it was labelled), testing and validation. The AIGG checklist also includes that the output or results of the AI should be made available within the patient’s electronic health record.

*Technical guidance* is provided by AI development expertise on the AIGG that is specific to the local IT context with an understanding of both its data storage and presentation systems. Technical guidance may also involve national or local standards, cyber security guidance and approval from local security officers as needed.

*Legal and contractual requirements* are predominantly concerned with the need for clear accountability and responsibilities, considering the health services’ role as guardians (kaitiakitanga) of our population’s health information. This includes legal obligations around privacy, such as the Health Information Privacy Code 2020^25^ and Code of Health and Disability Services Consumers’ Rights^26^. Specific considerations that have been developed include those around protection of the data should an AI company be sold, responsibility for ongoing monitoring and audit, accountability if an AI tool should fail, provisions for sharing IP and commercialisation, and conflicts of interest and how they may impact ongoing monitoring and evaluation.

## Discussion

Many frameworks and guidelines have been developed for AI internationally, however internal institutional review found that these were not appropriate for use in approving the translation of AI tools into clinical practice within the Waitematā healthcare service in Aotearoa NZ. This paper has presented the development of governance specific to our context and population. The resultant framework and checklist have been applied to proposals and projects at various points in the AI lifecycle – at the concept stage, model development and for the implementation of an externally developed AI tool. The AIGG asks that these initial projects report back regularly on their progress. In this way the AIGG will continue to learn from experience and the checklist will continue to evolve and be refined over time.

The review found that there were a number of key considerations that were insufficient or inappropriately framed in existing international frameworks for use in our District’s context. These were particularly focused around the importance of kaitiakitangi (guardianship) of people’s health information and recognition of Māori rights of data governance. This led to the establishment of a governance group that reflects the important stakeholders and decision-makers in our healthcare system, including consumers and Māori. To address these gaps, the AIGG developed a framework that includes specific considerations for Māori, consumers, equity, and ethics, all as separate issues.

The process for approval emphasises the importance of working with a range of end-user and expert groups, including Māori, consumers, clinicians, and services, so that development considers their perspectives and the operational realities of translating AI into clinical practice. Working with clinicians from the start will result in the development of AI tools that address clinical problems considered important by clinicians and as a result, will be more likely to be implemented in practice. Consideration of minority population groups and actively investigating the potential for bias is also important within the local context. Whilst these areas are potentially difficult for developers to address, the AIGG is able to provide connections, local knowledge of the data, ways to test for bias, and on-going plans for model monitoring. The overall process of the AIGG ensures that the relevant areas for AI development or implementation are systematically reviewed and potential risks identified.

One limitation of the AIGG’s approach is that it is a detailed time-consuming process. Being in the early stages of this journey, it is possible the review approach may be simplified once a better understanding of what is most important is ascertained. It could be argued that one national approach is entirely appropriate in a country with a relatively small population. The national health service (Te Whatu Ora) has established a national expert advisory group that will build on this work as well as the lessons learned from developing a COVID-19 algorithm governance framework^15^. The intention is to provide expert advice and assistance in the development and implementation of AI tools across the country. Further, there are some issues that still need to be addressed nationally, such as around accountability and indemnity, and regulation of software as a medical device. Alongside this a local governance process may also still be required to ensure local service involvement and appropriate approvals for data access.

It should be noted that Māori are under-represented in our health data due to issues related to service access for multiple reasons including mistrust of the health system, the inappropriateness of services and systematic racism. While Māori have been represented in our governance development process, it is possible that our work is not reflective of the wider Māori population.

Amongst the next steps being considered is the development of an AI Lab within Te Whatu Ora. The intention would be to bring together developers and academics with clinicians and consumers to collaborate on projects of interest to the health service. This could reduce some of the risk around data sharing and security, as the data would stay within the health service secure environment and governance. The AIGG is also planning a register of AI tools and communication with staff and the wider population on the governance process and the tools that have been reviewed.

These are the first steps taken in developing a practical process for the governance over the development and application of AI tools within the Aotearoa NZ health service context. The governance group and framework have been in place for some time now, however the effectiveness of this framework will likely only be apparent when the post-implementation reviews and audits of individual tools/projects become available. We acknowledge and embrace that our approach will adapt and evolve over time, reflecting changing contexts, increased understanding and experience, and improved methods.

## Methods

### Framework development

The governance framework was developed through background research on international guidance and best practices, patient surveys, analysis of internal data and software systems, integration with Māori data sovereignty principles^21^, and establishment of a representative governance group. The framework was further tested and refined by reviewing AI proposals.

Background research was conducted by the Te Whatu Ora Waitematā Institute for Innovation and Improvement (i3). This involved a review of the current state of AI implementation in clinical services internationally and recommendations around partnerships and contractual arrangements. National and international documents that were relevant in informing our processes are shown in Table 1.

**Table 1:** Useful background sources.

Source	Title	Description
International		
WHO	Ethics and Governance of Artificial Intelligence for Health^7^	Global guidance on what Ministries of Health need to consider for the governance of AI in Healthcare
NHSX	Artificial Intelligence: How to get it right^16^	An overview of the current data-driven technologies within the NHS healthcare system, highlighting current AI use-cases, and the policy required to ensure AI is utilised in a safe, effective and ethical manner.
	A Buyer’s Guide to AI in Health and Care^19^	A set of 10 questions required for making well-informed procurement decisions for AI systems.
Topol	The Topol Review – Preparing the healthcare workforce to deliver the digital future^20^	An independent review conducted for the NHS on key digital technologies, barriers to its adoption and potential solutions to these barriers.
Aotearoa NZ specific		
Hudson et al.	Te Ara Tika Guidelines for Māori Research Ethics^15^	Provides a framework for researchers to work with Māori ensuring research is undertaken based on tikanga and matauranga Māori (Māori ethical principles and philosophies).
	‘He Matapihi ki te Mana Rarauanga’ – Conceptualising Big Data through a Māori lens^21^	Provides concepts of Māori Data Sovereignty and presents a framework for conceptualising data/AI through tikanga and matauranga.
Te Mana Raraunga	Māori Data Sovereignty Charter and principles^22^	An outline of the concepts which underpin Māori Data Sovereignty.
NEAC	National Ethical Standards^23^	Guidelines developed to help foster awareness of ethical principles required for healthcare research in NZ.
Algorithm Charter Aotearoa and StatsNZ	Algorithm Charter for Aotearoa New Zealand^24^	A list of principles which governmental agencies within New Zealand have agreed to follow when developing or implementing AI systems.
Office of the Privacy Commissioner and StatsNZ	Principles for the Safe and Effective Use of Data and Analytics^25^	Six key principles for NZ’s government agencies to follow in order to support safe and effective analytics activities.
Social Wellbeing Agency.	Data Protection and Use Policy^26^	Policy statement and guidelines around collection and use of data or information.
StatsNZ	Algorithm Assessment Report 2018^27^	A cross-government review of how government agencies are using algorithms in NZ.

We conducted a cross-sectional survey and in-depth interviews to understand the perspectives of our healthcare service users regarding the secondary use of their personal health information for purposes such as AI. Inpatients and outpatients (*n* = 1377) were surveyed about what they expected the organisation was already doing with their health information and what their level of comfort was with secondary use including aggregation of data for service improvement and for the benefit of others^20^. The vast majority were comfortable with the aggregation of their data with others for the purposes of improving health care services for the future, with the conditions that it would produce benefit for others, their privacy would be maintained, data would still be secure, and appropriate governance or approvals were in place. The survey showed that generally people were comfortable with contributing the use of their health information for the greater good of the population, although better communication about this was requested. This includes transparency on projects undertaken and clarity around governance, confidentiality, and data security processes within our healthcare District. There were a small proportion of people uncomfortable with the use of their health information which was commonly linked to negative experiences with the health service.

The second phase of this research involved scenario based in-depth interviews (*n* = 12) including a scenario around the secondary use of data for AI development^27^. Participants reported conditional support for their health information being used for this purpose as it was advancing science and for the greater good. Participants reported that they needed to be able to trust their health service to respect these conditions. Conditions included adequate security and protection of the data, that the data was adequately de-identified and their privacy protected, that there was no potential for secondary harms, that there was good governance and clinical oversight, and lastly that the health information remained in the health system and was not shared with outside organisations or commercial companies. Where there was potential for commercial gains from the development of the AI, comfort levels decreased; participants described that in this case the intent might no longer be for public benefit.

A software engineering review was then conducted on the local database and software management systems. This review pointed out points of potential risk in terms of software development and maintenance, which are summarised in Table 2.

**Table 2:** Potential AI Risks as identified in our Software Engineering Review.

Risk type	Description
AI development risk	Risk of AI failing due to insufficient engineering due diligence in the development of the algorithm. Examples include but are not limited to: Direct coding errors, poor feature engineering, AI predicting on confounding features, absence of bias analysis, overfitting, poor problem definition.
Data accuracy risk	Risks associated with the accuracy of the data set. Examples include bias against data poor regions, bias through historical discrimination, unit errors, errors in data collection hardware, data labelling risks.
Software erosion risk	Risk of AI eroding overtime. This can be caused through classical software erosion such as system upgrades, or AI specific erosion such as changes in the healthcare environment (such as a pandemic), or feedback loop risk where a successful AI may distort patient outcomes in future datasets.
Clinical use risk	Risk of AI being used inappropriately or in a context where the intended health outcome is not able to be delivered

**Fig. 2:**
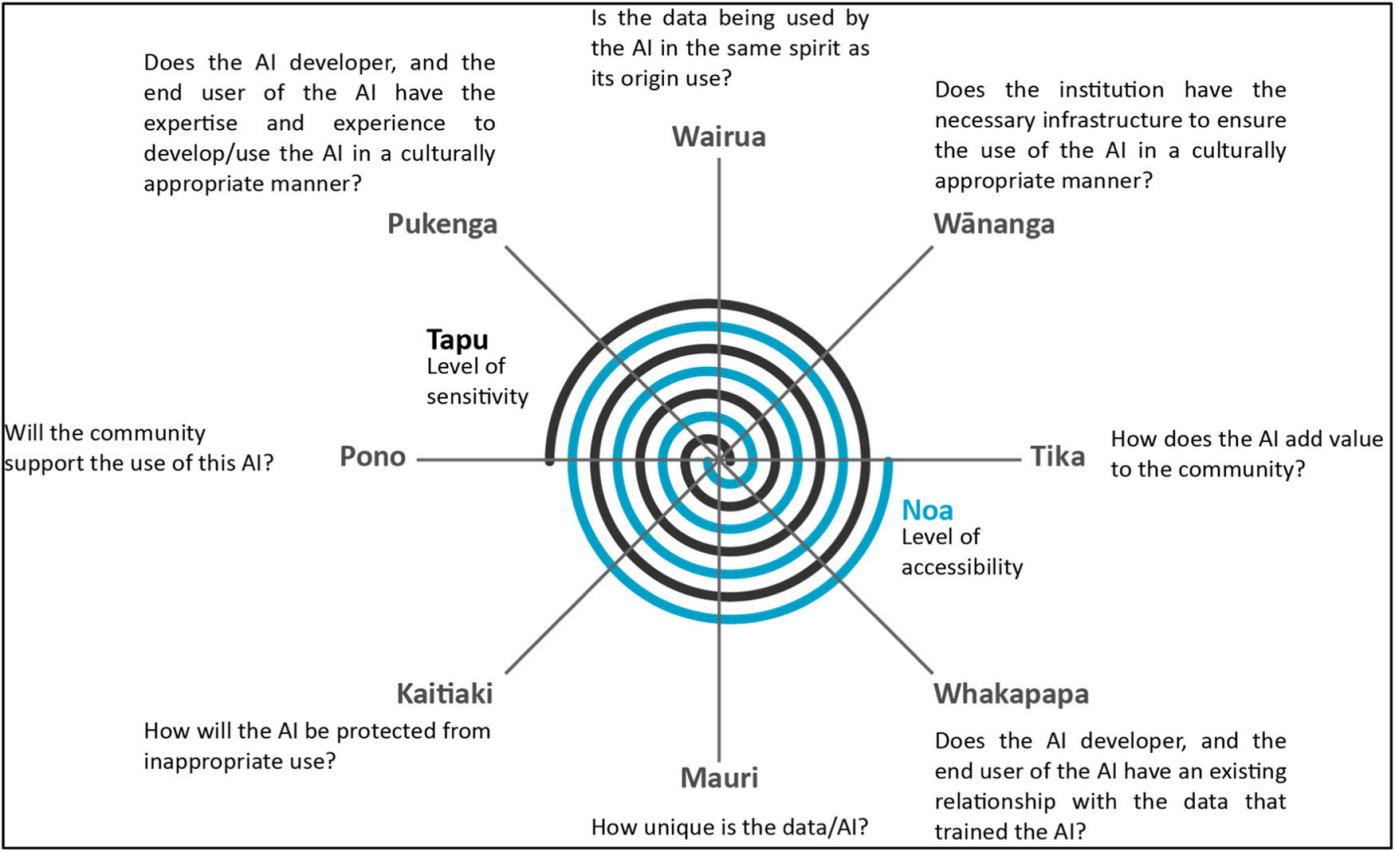
Te Mana o te Raraunga Framework consisting of two independent interwoven spirals. Adapted from^28^. Contains the following terms which are explained in the context of this figure only: • Whakapapa—used here to reflect the relationship between AI developer or the end user and the data required to develop the AI. • Mauri—relates to the level of originality associated with the data. • Kaitiaki—a term which describes guardianship of resources and in the context of AI, denotes the guardianship of patient data. • Pono—refers to the level of trust associated with the use of the data and includes the trustworthiness of the process and outcomes from using the data. • Pukenga—describes the knowledge or skill set of a person. • Wairua—relates to the spirit in which the data is being used. • Wānanga—reflects the level of responsibility associated with institutions that manage the data. • Tika—refers to the level of value associated with the use of the data. • Noa—reflects an assessment of the level of accessibility to the data. • Tapu—reflects an assessment of the level of sensitivity associated with the data.

A software framework was developed internally based on a previous exemplar framework to conceptualise big data through the lens of tikanga and matauranga Māori (Māori ethical principles and philosophies)^28^ and adapted for the AI context (Fig. 2). This framework provides a methodological process for evaluating AI, highlights potential areas of negative implications of AI for Māori, and creates the expectation that AI will be developed consistently with the key tenets of tikanga (ethical principles). In particular, it emphasises the obligations for layers of engagement with Māori necessary for a Te Tiriti honouring process, from concept to development, implementation and monitoring. This accountability to Māori has been expressed in the use of the takarangi (double helix spiral), building on the work of Te Ara Tika^18^ and He Matapihi ki te Mana Raraunga^28^, where one follows around the circumference asking the necessary questions to “make the road by walking”^29^. The notion of a spiral signifies that the questions are continually asked and reflected upon throughout the lifetime of the AI. For example, the second time the questions are asked will reveal a deeper understanding and further develop the capabilities of the stakeholders.

The next step was establishing a new AI Governance Group (AIGG) for Te Whatu Ora Waitematā. The Terms of Reference state that the purpose of the AIGG is to provide oversight and expert advice about the appropriateness, safety, effectiveness, ethics and ongoing improvement of any AI research, development, projects, partnerships, contracts, or implementation at Waitematā. It was considered vital that the following areas of representation be included: consumers, clinical governance, data and digital governance, privacy and security, legal, Māori health, Pacific Island health, research, analytics, innovation and improvement (supporting implementation), and external expertise in AI and machine learning.

**Fig. 3:**
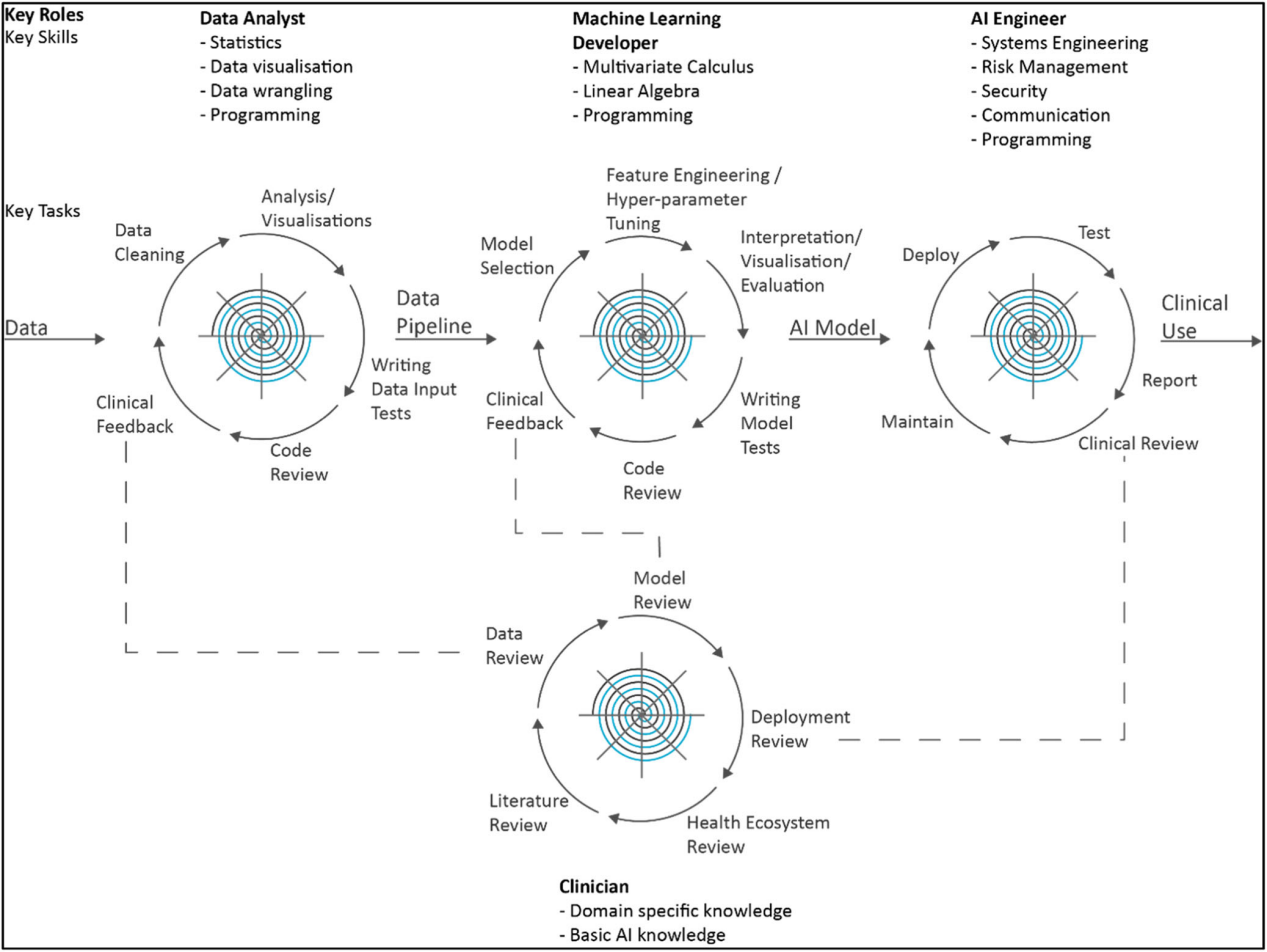
The AI Development Lifecycle: The AI development workflow consisting of four key roles. The tasks are cyclic and grounded within the takarangi of the key tenets of tikanga as described in Fig. 2.

The final step was adapting all of the above into a checklist for use by the AIGG when considering new proposals for access to data or clinicians at any stage of the AI development workflow from development to implementation (Fig. 3).

### Initial testing of the framework

Following development of the framework, testing was undertaken by the AIGG reviewing proposals for AI tools intended to be used within the health service. These included a tool for identifying the potential early signs of diabetic retinopathy on retinal screening images, and the early development of a COVID risk of hospitalisation score. Through initial testing, the AIGG identified particular issues to be addressed which allowed us to further refine the checklist. Some examples of considerations added after the first iteration include conflicts of interest (of clinicians who are also developers/ entrepreneurs), ongoing monitoring and accountability, responsiveness to potential future changes in ownership and accountability, and the ability to share benefits with the public health system on behalf of the Waitematā population.

### Māori terms

A number of Māori language terms are included in this description of methods. These are explained here:

- Tino rangatiratanga—the sovereign right for Māori to be in charge of their own resources and aspirations, acting with authority and independence over their own affairs.
- Tikanga—Māori customary practices and behaviours.
- Taonga—an object or resource which is viewed by Māori as a treasured possession.
- Kaitiakitanga—describes guardianship and protection based upon the Māori world view.

### Reporting summary

Further information on research design is available in the Nature Research Reporting Summary linked to this article.

### Supplementary information

Reporting Summary

Supplemental Material

## Data Availability

Relevant data are available from the authors as appropriate.
