# Supplementary material for: An example of governance for AI in health services from Aotearoa New Zealand
Source: NPJ Digit Med. 2023 Sep 1;6:164. doi: 10.1038/s41746-023-00882-z (PMC10474148; doi:10.1038/s41746-023-00882-z)
Supplement: Supplementary file 2 — Supplemental Material [file 41746_2023_882_MOESM2_ESM.pdf]

AI Governance Checklist Questions at Points in the AI Lifecycle

AI Lifecycle	Concept development	Access to data for pre-processing, labelling, or model development	Validation or implementation of an existing AI model
Appropriateness	Is the problem they are trying to solve understood?<br><br>Is that problem associated with a large quantity of data which an AI model could learn from?<br><br>Would analysis of that data be on a scale so large and repetitive that humans struggle to carry it out effectively?	Is the problem they are trying to solve understood and could the AI offer advantages over what is currently provided?<br><br>Is that problem associated with a large quantity of data which an AI model could learn from?<br><br>Would analysis of that data be on a scale so large and repetitive that humans struggle to carry it out effectively?	Is the problem in our context understood and does the AI offer benefits over the current situation?<br><br>Has it been sufficiently tested for accuracy against empirical evidence?<br><br>Has it been used in practice elsewhere and does it perform as expected?<br><br>Has it been, or how will it be, sufficiently validated with data from the NZ population?<br><br>How will its impact be evaluated?
Consumers/ population perspectives	Would the community support the use of data/AI for this problem?<br><br>What difference would it make to the community/population?<br><br>Are there any risks to wellbeing and safety? Who bears the risk (is it the same people who are likely to benefit from the project)? Can they potentially be mitigated?	Would the community support the use of data for this problem?<br><br>What difference would this AI make to the community/population?<br><br>Are there any risks to wellbeing and safety? Who bears the risk (is it the same people who are likely to benefit from the project)? How will they be mitigated?<br><br>Has the project engaged with people/groups who may be impacted by the use of this AI?	Would the community support the use of data/AI for this problem?<br><br>What benefits does it bring to the community?<br><br>Has the project engaged with people/groups who may be impacted by the use of this AI?<br><br>Would we need to communicate with our patients/ population about this use of AI?<br><br>Are there any risks to wellbeing and safety? Who bears the risk (is it the same people who are likely to benefit from the project)? How will these be avoided or mitigated?<br><br>Do our patients need to have this use of AI communicated with them?
Māori perspectives	Are those involved able to design this AI in a culturally appropriate manner?<br><br>Does the project adhere to the relevant Te Mana Raraunga principles and Te Ara Tika principles (acceptability and accountability to Māori, relational ethic to working with Māori, equitable benefits through focus on mana, equity and distributive justice)?<br><br>Does the project embed a te ao Māori perspective through a te tiriti based partnership approach?	Are those involved able to develop this AI in a culturally appropriate manner?<br><br>Has the team engaged with Māori or had any Māori governance input/oversight?<br><br>Are there any potential issues from a te ao Māori perspective?	Was the AI developed in a culturally appropriate manner?<br><br>Has the development and validation had any Māori governance oversight or input from Māori? Have any concerns been raised and addressed?

Equity and Fairness	Are there likely to be any bias or discrimination issues with addressing this problem using the available data?	Are there likely to be any bias or discrimination issues with addressing this problem using the available data?<br><br>Have any fairness issues been identified to date and are there measures to mitigate risks arising from potential bias?	Has it been evaluated for bias across ethnic groups, genders, other?<br><br>Can it be used fairly?<br><br>Have any fairness issues been identified to date and are there measures to mitigate risks arising from potential bias?
Ethical principles	Is the data able to be used ethically and safely and in the spirit within which was collected?<br><br>Does the project comply with the NEAC standards?<br><br>Does the project intend to embed the ethical principles, such as transparency and human autonomy? Does the team agree to transparency of their methods and model?	Is the data able to be used ethically and safely and in the spirit within which was collected?<br><br>Does the project comply with the NEAC standards?<br><br>Does the project intend to embed the ethical principles, such as transparency and human autonomy?<br><br>Is there intended to be human oversight of the AI and how will that occur?<br><br>Does the project intend to comply with our principle of transparency?	Can the AI be used ethically? Are the ethical principles embedded in its development and use?<br><br>Have they disclosed the AI for transparency?<br><br>Will a human still be involved in oversight/final decisions?<br><br>Does the project comply with the NEAC standards?
Clinical perspectives	Could the outputs of a model be tested for accuracy against empirical evidence?<br><br>Would model outputs lead to problem solving in the real world from a clinical perspective?	Can the outputs of such a model be tested for accuracy against empirical evidence?<br><br>Will model outputs lead to problem solving in the real world from a clinical perspective?	Can the AI be implemented in our clinical workflow and operational context?<br><br>Will it be accepted and explainable/understood by clinicians?<br><br>Is there clinical and service support in our DHB?<br><br>Has there been an assessment of feasibility/readiness, communication and training?<br><br>Who is responsible for ongoing audit and monitoring from a clinical perspective?
Data availability, quality, appropriate-ness, and completeness	Is the necessary data available?<br><br>Is the data of sufficient quality and completeness?<br><br>Is there any further consent required for this use of the data?	Is the problem associated with a large quantity of data in our DHB which an AI model could learn from?<br><br>Is the necessary data available in the manner needed to run the AI?<br><br>Is the data of sufficient quality and completeness?<br><br>Is any further consent required for the use of the data?<br><br>If data is provided, is there a plan for storage, destruction or retention?<br><br>Are there any risks around privacy, confidentiality, security or re-identification?	Is it clear what data the model was developed with and then tested on?<br><br>What data has/will it be validated with? Is that data of sufficient quality and completeness?<br><br>Is it an appropriate use of the data according to how the data was collected in our DHB?<br><br>Are there risks around data privacy, confidentiality, security, or re-identification?<br><br>Will data and/or results from the model come into our data warehouse?

Technical processes	Are those involved able to design according to the DHBs technical specifications?	Are those involved able to develop and test according to the DHBs technical specifications?<br><br>Is the data and process safeguarded with appropriate security and confidentiality?<br><br>Does the team agree to transparency of their processes, data, methods and models?	Have they disclosed the AI so that it can be evaluated technically – model chosen and methodologies, feature engineering, parameter tuning?<br><br>Does it comply with our technical specifications?<br><br>Who is responsible for ongoing audit and monitoring from a technical perspective?
Contractual and Legal issues	Can we agree on a co-design contract with respect to DHB principles, confidentiality, accountability, and shared IP?<br><br>Have the necessary approvals been given (Research & Learning, HDEC etc)?	Can we agree on a contract with respect to our principles, privacy/confidentiality, security, Māori data sovereignty, IP, accountability, publication and commercialisation plans?<br><br>Have the necessary approvals been given (Research & Learning, HDEC etc)?	Can we agree on a contract with respect to DHB principles, use of data and outputs, privacy/confidentiality, Māori data sovereignty, ongoing monitoring and audit, regular review processes?<br><br>Is accountability/liability between parties clear and understood?<br><br>What will happen should the AI fail?
